# Effect of aerobic exercise against vanadyl sulphate-induced nephrotoxicity and hepatotoxicity in rats

**DOI:** 10.15171/jrip.2016.39

**Published:** 2016-08-12

**Authors:** Fatemeh Ahmadi, Mehdi Nematbakhsh, Mehdi Kargarfard, Fatemeh Eshraghi-Jazi, Ardeshir Talebi, Soheila Shirdavani

**Affiliations:** ^1^Water & Electrolytes Research Center, Isfahan University of Medical Sciences, Isfahan, Iran; ^2^Department of Sport Physiology, University of Isfahan, Isfahan, Iran; ^3^Department of Physiology, Isfahan University of Medical Sciences, Isfahan, Iran; ^4^IsfahanMN Institute of Basic & Applied Sciences Research, Isfahan, Iran

**Keywords:** Aerobic exercise, Vanadyl sulphate, Hepatotoxicity, Nephrotoxicity, Rat

## Abstract

**Introduction:** Vanadium compounds are insulin like drugs which are accompanied with nephrotoxicity and hepatotoxicity as their major side effects. Aerobic exercise is well known as an approach to reduce the side effects of many drugs.

**Objectives:** This study was designed to determine the role of aerobic exercise against vanadyl sulphate induced nephrotoxicity and hepatotoxicity in male rats.

**Materials and Methods:** Twenty-four male Wistar rats were randomly divided into three groups. Group I had aerobic exercise on a treadmill 5 days/week for 6 weeks. Group II received vanadyl sulphate (50 mg/kg/week; i.p.) for 6 weeks. Group III had combination of exercise and vanadyl sulphate therapy as groups 1 and 2. At the end of study, blood samples were obtained, and the animals were sacrificed for the tissues injury determination.

**Results:** Vanadyl sulphate alone increased serum levels of blood urea nitrogen (BUN), creatinine (Cr), and kidney weight (KW) and kidney tissue damage score (KTDS) (*P*<0.05). These observations revealed nephrotoxicity induced by vanadyl sulphate, although exercise training did not attenuate these results. In addition, vanadyl sulphate alone induced liver tissue damage score and exercise training intensified it insignificantly, while the serum levels of aspartate amino transferase and alanine amino transferase were greater in exercise alone group than others groups.

**Conclusion:** Aerobic exercise could not attenuate vanadyl sulphate induced nephrotoxicity and hepatotoxicity. These findings must be considered when vanadyl sulphate is suggested as insulin like drug.

**Implication for health policy/practice/research/medical education:**

Aerobic exercise could not attenuate vanadyl sulphate induced nephrotoxicity.

## Introduction

Vanadium is a trace element which is considered to be essential for biological processes and it has been shown that vanadium compounds could improve or prevent diabetes symptoms in practical models (1). Vanadium remains in different organs such as kidney and liver even several months after treatment withdrawal (2), and induces toxic effects in liver and kidney (3). Vanadium increases hepatoxicity by increasing reactive oxygen species (ROS) and induces functional and structural changes in kidney (4-6). Physical activity can be used as an excellent tool for the reduction and treatment of many diseases such as diabetes and cardiovascular diseases (7). It is documented that exercise has protective effect against hepatotoxicity induced by doxorubicin in rats (8). Also, lifestyle including exercise improves chronic progressive liver disease in obese individuals (9). In addition, some studies showed effect of exercise on renal function (10,11), and it may protect kidneys against renal ischemia/reperfusion (12), and attenuate renal damage induced by nitric oxide deficiency in animal model (13).

The data were reported as mean ± SEM. * and # indicate significant difference from the EX and Va groups, respectively (*P*<0.05). Abbreviations of Ex, Va and Ex+Va were used stand of exercise, vanadyl sulphate and the combination of exercise and vanadyl sulphate.

## Objectives

This research was designed based on this goal that whether aerobic exercise on treadmill can ameliorate hepatotoxicity and nephrotoxicity induced by vanadyl sulphate administration in rat animal model or not.

## Materials and Methods

### Experimental protocol

Twenty-four adult male (weighing 203.5±5.63 g) Wistar rats (Animal Center, Isfahan University of Medical Sciences, Isfahan, Iran) were used in this research. The animals had free access to water and rat chow and were housed at a temperature of 23–25°C under a 12-h light/ 12-h dark schedule. All rats were randomly assigned to three groups. The animals in group I (called Ex group) had aerobic exercise on a treadmill 5 days/week for period of 6 weeks. Group II (called Va group) received vanadyl sulphate (50 mg/kg/week; i.p.) for period of 6 weeks. Group III (called Va+Ex group) was subjected to physical activity and vanadyl sulphate therapy as groups 1 and 2.

### Treadmill exercise

Animals were habituated to treadmill running for 5 days (10 min/day at 0% grade). Rats quickly learned to stay on the belt and avoid shock. After end of familiarization period, the animals were forced to run on treadmill at speed of 17 to 20 m/min (zero slope) for 30 to 55 min/session, 5 days/week for period of 6 weeks (8,14) (Table 1).

**Table 1:** Exercise training protocol

**Week**	**1**	**2**	**3**	**4**	**5**	**6**
Time (min/session)	30	35	40	45	50	55
Speed(m/min)	17	17	18	18	20	20

### Measurement and histopathological procedures

At the end of the experiment, the rats were anesthetized with chloral hydrate injection (450 mg/kg; i.p.) and blood samples were taken from the heart. Then, the animals were sacrificed and kidneys and sections of the liver were removed and weighed. The blood samples were centrifuged at 6000 g. Then, serum was collected and stored at -20°C until measurement. The right kidney and a section of liver were homogenized and centrifuged at 15000 g. Then, the supernatant was stored at -20^°^C for measurement. The serum levels of creatinine (Cr), blood urea nitrogen (BUN) and liver enzymes (aspartate amino transferase [AST], alanine amino transferase [ALT]) were determined using quantitative diagnostic kits (Pars Azmoon, Iran) by automatic analyzer (Technicon, RA1000 model). The serum, kidney and liver levels of nitrite (stable NO metabolite) were measured using a colorimetric assay that involves the Griess reaction. The serum, renal and liver levels of malondialdehyde (MDA) were measured manually.

The left kidney and other section of the liver were fixed in 10% neutral formalin solution and embedded in paraffin. Slices were stained by hematoxylin and eosin staining method. Kidney tissue damage score (KTDS) and liver tissue damage score (LTDS) were blindly investigated by a pathologist. Liver and kidney tissue damage score was graded from 1 to 4 based on the intensity of lesions. Score of zero was assigned for normal tissue.

### Ethical issues

Prior to the experiment, the protocols were confirmed to be in accordance with the Guidelines of Animal Ethics Committee of Isfahan University of Medical Sciences. The experimental procedures were approved in advance by the Isfahan University of Medical Sciences Ethics Committee. (Ethics committee # IR.MUI.REC.1393.2.360).

### Statistical analysis

Data were expressed as mean±SEM. One-way analysis of variance (ANOVA) followed by LSD was applied to compare the BUN, Cr, nitrite, ALT, AST and MDA levels; and kidney weight (KW) between the groups. To compare KTDS and LTDS the between the groups, Kruskal-Wallis and Mann-Whitney U tests were applied. Normalized body weight was analyzed by repeated measured analysis method. *P*<0.05 was considered statistically signiﬁcant.

## Results

### Effect of exercise and vanadyl sulphate on serum levels of BUN and Cr

**Figure 1:**
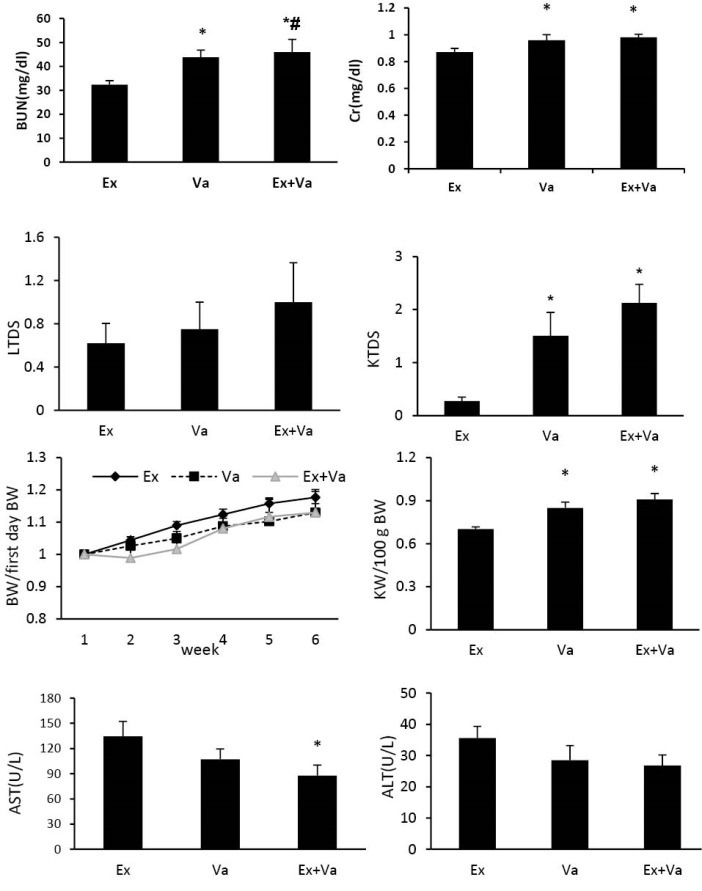
The serum levels of blood urea nitrogen ( BUN), creatinine (Cr), aspartate amino transferase (AST), alanine amino transferase (ALT), kidney tissue damage score ( KTDS), liver tissue damage score (LTDS), kidney weight (KW) g/100 g body weight (BW), normalized body weight (NBW) in experimental groups.

Administration of vanadyl sulphat**e** increased the serum levels of BUN and Cr when compared with Ex group (*P*<0.05). Exercise accompanied with vanadyl sulphat**e** intensified the serum level of BUN in comparison with Va group (*P*<0.05) (Figure 1).

### Effect of exercise and vanadyl sulphate on levels of MDA, nitrite, AST and ALT

The serum level of nitrite in Ex+Va group was decreased significantly in compared with Va group (*P*<0.05). Exercise also increased the serum and liver level of MDA in Ex+Va significantly when compared with Va group (*P*<0.05). No significant differences were observed in liver and kidney levels of nitrite and kidney level of MDA between the groups (Table 2). The serum levels of AST (significantly) and ALT (insignificantly) decreased in Ex +Va group compared with Ex group (Figure 1).

**Table 2:** Levels of nitrite (N) and malondialdehyde (MDA) in serum (S), kidney (K) and liver (L)

**Groups**	**Parameters**					
	**SN (µmole/L)**	**KN (µmol/g tissue)**	**LN (µmol/g tissue)**	**SMDA (µmole/L)**	**KMDA (nmol/g tissue)**	**LMDA (nmol/g tissue)**
Ex	17.53 ± 2.98	0.32 ± 0.04	0.16 ± 0.01	1.34 ± 0.12	1.01 ± 0.15	3.46 ± 0.29
Va	14.18 ± 1.85	0.27 ± 0.03	0.21 ± 0.02	1.31 ± 0.12	1.50 ± 0.31	3.62 ± 0.22
Va+Ex	9.84 ± 1.17*	0.30 ± 0.05	0.21 ± 0.02	1.98 ± 0.33*#	1.53 ± 0.19	4.83 ± 0.77*

The data were reported as mean ± SEM. Abbrivations of Ex, Va and Ex+Va were used stand of exercise, vanadyl sulphate and the combination of exercise and vanadyl sulphate. * and # signs stand for significant difference from Ex or Va group respectively (*P*<0.05).

### Effect of exercise and vanadyl sulphate on KTDS, LTDS, KW and body weight

**Figure 2:**
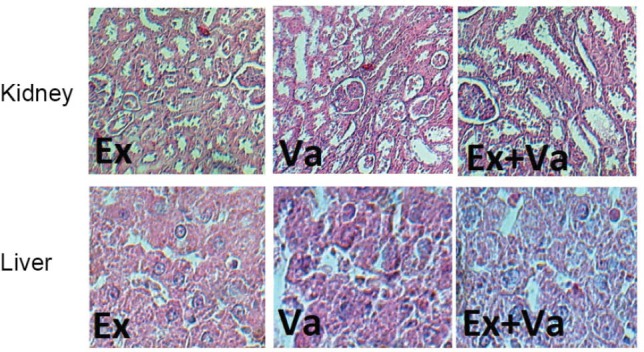
The images of kidney and liver tissues (magnification ×100) in experimental groups. Abbreviations of Ex, Va and Ex+Va were used stand of exercise, vanadyl sulphate and the combination of exercise and vanadyl sulphate.

Vanadyl sulphate increased KW , KTDS (significantly; *P*<0.05) and LTDS (insignificantly) when compared with group Ex and exercise accompanied with vanadyl sulphate could not ameliorate these changes. BW was increased during the experiment in all three groups with no significant differences between the groups (Figure 1). The samples of image for kidney and liver are shown in Figure 2.

## Discussion

The main objective of this investigation was to test the role of aerobic exercise on vanadyl sulphate induced nephrotoxicity and hepatotoxicity in rat. The results showed that administration of vanadyl sulphate induced nephrotoxicity that were not attenuated by physical activity. In agreement with our findings, some studies have shown that vanadium compounds disturbed renal function and induced histology changes (4-6). An evidence showed that vanadyl sulphate aggravated nephrotoxicity induced by cyclosporine A in diabetic rats (15). Vanadium compounds induces hypokalemic distal renal tubular acidosis which is related to inhibiting H(+)-K(+)-ATPase activity (16). Vanadium caused increasing urinary excretion of water and salts and affected renal Na(+)-K(+)-ATPase (17). Vanadium accumulates in renal tissue (4) and increases lipid peroxidation (5) and collagen content (6) in kidney. The present study showed that exercise training not only could not ameliorate nephrotoxicity induced by vanadyl sulphate, but also aggravated the damage. It was documented positive effects of exercise in literature (7-9), and it improved glomerulosclerosis in hypertensive model (18) and protected the kidney against renal ischemia-reperfusion injury (12). In agreement with us, one study revealed that exercise training could not affect kidney function against chronic renal failure (19). In addition, regular exercise aggravated renal damage in hypertensive animals (20). Exercise also had adverse effect on immune complex-mediated glomerulonephritis in rabbit animal model (21). In current study, administration of vanadyl sulphate alone decreased serum level of ALT, AST enzymes. According with our results, Koyuturk et al (22) showed that administration of vanadyl sulphate decreased serum levels of AST, ALT, and ALP in diabetic animals. Also, recent study revealed decreasing in serum levels of ALT, and ALP by vanadyl sulphate in normal animals, however these changes were not significant (22). Also, pathological findings demonstrated that exercise intensified LTDS induced by vanadyl sulphate, although it was not significant. It seems that there is a correlation between ascending enhancement of LTDS and descending decreasing of liver enzymes. Koyuturk et al (22) reported that administration of vanadyl sulphate induced lipid peroxidation in normal rat liver.

In this study exercise accompanied with vanadyl sulphate reduced serum level of nitrite. In addition, exercise training affects serum nitrite level in young men (23). Nitric oxide (NO) is synthesized by NO synthases (NOS) from L-arginine. Three different isoforms of NOS are known including the neuronal NOS (nNOS/NOS-1), inducible NOS (iNOS/NOS-2), and endothelial NOS (eNOS/ NOS-3) (24). Perhaps in this study eNOS production was affected on serum level of nitrite.

Vanadyl sulphate accompanied with exercise increased serum and liver MDA level. Liu et al (25) reported that dietary vanadium intake induced renal and hepatic toxicity through oxidative damage and vanadium accumulation. Koyuturk et al reported that vanadyl sulphate enhanced liver MDA level in normal rats (22).

In this study, exercise training did not exhibit beneficial effects against kidney and liver damage induced by vanadyl sulphate, even it tended to aggravate tissue damage in both liver and kidney, however exercise training protocol used in this study had presented cardioprotective and hepatoprotective effects (8,14). We supposed that exercise increased blood flow into internal organs such as kidney and liver which was resulted in absorption‏ and‏ accumulation of‏ vanadium in these organs and advanced into further damage.

## Conclusion

It was concluded that, not only exercise training did not ameliorate kidney and liver damage induced by vanadyl sulphate, but also it tended to aggravate renal and liver damage. Therefore, these findings are the disadvantage of vanadyl sulphate to be candidate as insulin like drug.

## Authors’ contribution

FA, FEJ and SS conducted the research. MN designed and supervised the study, analyzed the data and prepared the final draft of the article. MK designed and supervised the study. AT supervised and analyzed the pathology data.

## Conflicts of interest

The authors declared no competing interests.

## Ethical considerations

Ethical issues (including plagiarism, data fabrication, double publication) have been completely observed by the authors.

## Funding/Support

This research was supported from Isfahan University of Medical Sciences (Grant #293360).
